# Dual Burden of Food and Water Insecurity Among SNAP Households with Children in the Southern United States

**DOI:** 10.3390/ijerph23070891

**Published:** 2026-07-10

**Authors:** Nila Pradhananga, Jean Pierre Enriquez, Harriet Okronipa, Denise Holston, Jeffrey M. Sadler

**Affiliations:** 1 Department of Nutritional Sciences, Oklahoma State University, Stillwater, OK 74075, USA; harriet.okronipa@okstate.edu; 2 Department of Nutrition, College of Agriculture, Biotechnology & Natural Resources, University of Nevada, Reno, Las Vegas, NV 89123, USA; jeanenriquez@unr.edu; 3 School of Nutrition and Food Sciences, Nutrition and Community Health, LSU AgCenter, Louisiana State University, Baton Rouge, LA 70803, USA; dholston@agcenter.lsu.edu; 4 Department of Biosystems and Agricultural Engineering, Oklahoma State University, Stillwater, OK 74075, USA; jeff.sadler@okstate.edu

**Keywords:** food insecurity, water insecurity, HWISE, SNAP, households with children, Southern United States, health disparities, social determinants of health

## Abstract

**Highlights:**

**Public health relevance—How does this work relate to a public health issue?**

- Food insecurity and water insecurity are critical yet often separately examined social determinants of health that disproportionately relate to low-income households in the United States (U.S.); their co-occurrence represents a compounded burden with implications for health equity and well-being.
- Supplemental Nutrition Assistance Program (SNAP) households with children in the Southern U.S. face heightened vulnerability due to intersecting socioeconomic disadvantage, environmental stressors, and infrastructure inequities, positioning them at increased risk of multiple, overlapping resource insecurities.

**Public health significance—Why is this work of significance to public health?**

- This study provides empirical evidence of a substantial co-occurrence of food and water insecurity, with nearly half of households experiencing both simultaneously, extending existing research by quantifying this dual burden among SNAP households with children in the Southern U.S.
- The strong association observed between food insecurity and water insecurity suggests that these conditions reflect interconnected dimensions of material hardship, highlighting the limitations of assessing food insecurity in isolation when evaluating household-level vulnerability.

**Public health implications—What are the key implications or messages for practitioners, policy makers, and/or researchers in public health?**

- Public health practitioners and policy makers should adopt integrated, multisector approaches that address both food and water access, recognizing their combined influence on health outcomes and the need for coordinated intervention strategies.
- Incorporating water insecurity into routine surveillance systems may improve identification of high-risk households and support more equitable and effective resource allocation.

**Abstract:**

**Background:** Food insecurity and water insecurity are increasingly recognized as interconnected social determinants of health; however, their co-occurrence remains underexplored in U.S. populations. SNAP households with children experience a high prevalence of both food and water insecurity. This study estimated the prevalence of food insecurity and water insecurity and examined their co-occurrence among SNAP households with children in the Southern U.S. **Methods:** A cross-sectional, web-based survey was conducted among 683 SNAP participants residing in households with children. Food insecurity was assessed using the 10-item USDA Adult Food Security Survey Module, and water insecurity was measured using the Household Water Insecurity Experiences (HWISE) scale. Descriptive statistics estimated prevalence, and regression analyses assessed associations. **Results:** Food insecurity (75.1%) and water insecurity (53.9%) were highly prevalent among surveyed SNAP households with children. Nearly half of households (48.6%) experienced both conditions, while 19.6% were secure in both. Food insecurity alone was reported by 26.5% of households, and water insecurity alone by 5.3%. Higher food insecurity scores were associated with increased odds of water insecurity (AOR = 1.33, 95% CI: 1.26–1.40, *p* < 0.001). **Conclusions:** Food insecurity and water insecurity frequently co-occur among SNAP households with children. Integrated public health strategies addressing both food and water access are needed to reduce disparities and improve household well-being.

## 1. Introduction

Water is fundamental to physical health, nutrition, and psychosocial well-being; however, challenges related to its availability, accessibility, reliability, and safe use continue to pose significant risks to health and human development globally [1,2]. In the United States (U.S.), these challenges are not evenly distributed. Public water supplies are often unavailable to economically disadvantaged households, resulting in reliance on unregulated private wells or alternative water sources. Such conditions limit access to safe and reliable drinking water, increasing exposure to environmental contaminants and associated health risks [3,4,5]. Although water insecurity is often overlooked in high-income countries, studies have documented substantial water insecurity among socioeconomically vulnerable U.S. populations, particularly in rural, low-income, and historically underserved communities. These disparities are particularly pronounced in low-income rural and minority communities, where historical underinvestment in public infrastructure has contributed to persistent inequities in water access. As a result, many households must depend on costly or potentially unsafe alternatives, including hauled water, bottled water, or small-scale systems, further exacerbating economic and health burdens [6,7]. Household characteristics are important determinants that significantly influence the degree of household water insecurity [8]. Despite the central role of water in sustaining life and supporting food systems, the measurement of water insecurity has lagged behind that of other resource insecurities (e.g., food insecurity) [7]. While food insecurity has long been systematically defined and monitored, water insecurity remains less consistently conceptualized and measured, particularly in high-income settings such as the U.S. This imbalance has limited the ability to fully understand the scope, drivers, and consequences of inadequate water access at the household level.

In contrast, the field of food security offers well-established measurement approaches [9]. Food security is defined as a condition in which all individuals, at all times, have physical and economic access to sufficient, safe, and nutritious food to meet their dietary needs and preferences for an active and healthy life [10]. The development of household and individual food insecurity metrics has been highly influential, enabling researchers to identify at-risk populations, allocate scarce resources more effectively, and uncover previously overlooked and significant consequences of food insecurity [11]. In the U.S., food insecurity is routinely assessed using standardized tools such as the U.S. Department of Agriculture (USDA) Food Security Survey Modules [12]. Recent national estimates indicate that 13.7% of U.S. households experienced food insecurity in 2024, representing approximately one in seven households [13]. Food insecurity is more prevalent among households with children, affecting nearly one in five such households. Among households with children, 9.1% reported food insecurity among children, indicating that children themselves were directly affected by inadequate food access [13]. These figures underscore persistent socioeconomic disparities in access to adequate nutrition, particularly among low-income populations and those participating in federal assistance programs such as the Supplemental Nutrition Assistance Program (SNAP). Although food insecurity measurement frameworks capture economic and physical access to food, they do not explicitly account for water access, despite water being essential for food safety, preparation, and dietary quality [14]. This omission is critical, as water and food insecurities are inherently interconnected and may co-occur within the same households, compounding risks to health and well-being [15,16]. The concept of a “dual burden” of food insecurity and water insecurity reflects not only their overlap but also their potential to interact synergistically, amplifying adverse nutritional, psychosocial, and health outcomes [14,17,18].

Efforts to address this gap have led to the development of the Household Water Insecurity Experiences (HWISE) scale, a cross-culturally validated instrument designed to measure household-level water insecurity across diverse settings [19,20]. Developed using data from thousands of households across multiple countries, the HWISE scale captures key dimensions of water insecurity, including availability, access, reliability, and use, and has been shown to produce comparable and reliable estimates across contexts [20]. The HWISE scale has generated important global health insights by enabling consistent assessment of water insecurity at the household level.

These impacts are particularly pronounced in households with children, where water insecurity has been shown to affect infant feeding practices through multiple pathways, including feeding frequency, food hygiene, and dietary diversity [21]. Caregivers also associate water-related challenges with adverse child health outcomes, such as increased susceptibility to infectious diseases, undernutrition, and mortality. The Southern U.S. is particularly vulnerable to drought and remains economically and socially disadvantaged, increasing susceptibility to resource insecurity [22,23]. Water insecurity and food and nutrition insecurity are deeply interconnected; however, relationships remain underexplored and insufficiently understood [24]. Despite growing global evidence, water insecurity remains insufficiently examined in U.S. populations, particularly among vulnerable groups [25]. This gap is especially salient for SNAP-participating households with children, who already face elevated risks of food insecurity and may simultaneously experience constraints related to water access. In this context, the present study applies the Household Water Insecurity Experiences (HWISE) scale and the United States Department of Agriculture (USDA) Food Security Survey Module to assess the dual burden of food and water insecurity among SNAP households with children in the Southern U.S. We hypothesized that food insecurity would be positively associated with water insecurity and that a substantial proportion of households would experience both concurrently.

## 2. Methods

### 2.1. Study Design and Sample

This study utilized a cross-sectional design based on data collected through a web-based survey administered via a Qualtrics research panel. Eligible participants were adults aged 18 years or older who reported receiving SNAP benefits within the previous 12 months and resided in the Southern U.S. Census region, including the District of Columbia. For the present analysis, the inclusion criteria were restricted to respondents living in households with at least one child under the age of 18 years. Participants were recruited through Qualtrics using panel-based sampling methods, and invitations containing a survey link were distributed electronically. All respondents provided informed consent before participation. The survey was anonymous, and no personally identifiable information was retained. The study protocol was reviewed and classified as exempt by the Oklahoma State University Institutional Review Board (IRB-25-532).

### 2.2. Measure

Participants self-reported demographic and household characteristics, including age, gender, race/ethnicity, educational attainment, employment status, and household income. Household composition variables included total household size and the number of children under 18 years. Household food insecurity over the past 12 months was assessed using the validated 10-item U.S. Department of Agriculture (USDA) Adult Food Security Survey Module [12]. Responses were summed to generate a total score ranging from 0 to 10, with higher scores indicating greater severity of food insecurity. Consistent with established scoring guidelines, respondents were categorized into food-secure (scores 0–2) and food-insecure (scores 3–10) groups for analytic purposes [12]. Household water insecurity was measured using a brief, validated, 4-item Household Water Insecurity Experiences Scale (HWISE), which includes worry, changed plans, insufficient drinking water, and inability to wash hands, which captures challenges related to the availability, reliability, and adequacy of water access [19,20]. Items assessed experiences such as insufficient water for daily needs and concerns about water access. Respondents reported the frequency of each experience over the previous four weeks using standardized response categories (never = 0, rarely = 1, sometimes = 2, often/always = 3). Responses were summed to generate a composite score ranging from 0 to 12, with higher scores indicating greater water insecurity. Households with scores ≥ 4 were classified as having moderate-to-high water insecurity, while those with scores < 4 were classified as water-secure/low water insecurity [20]. To examine the co-occurrence of food and water insecurity, a four-category variable was created: (i) food- and water-secure, (ii) food-insecure only, (iii) water-insecure only, and (iv) dual burden of food and water insecurity.

### 2.3. Analysis

All statistical analyses were conducted using SPSS (Version 30). Descriptive statistics were calculated to summarize participant characteristics. Categorical variables were reported as frequencies and percentages. The prevalence of food insecurity, water insecurity, and their co-occurrence was estimated using descriptive analyses. Covariate adjustment was performed in the logistic regression model, which served as the primary multivariable analysis. Logistic regression results were reported as odds ratios (ORs), 95% confidence intervals (CIs), and *p*-values. In addition, a binary logistic regression model was used to examine the association between food insecurity score and water insecurity status, adjusting for household size and number of children. Household size and number of children were selected a priori because they directly influence household resource demands and have been previously associated with food and water insecurity. The food insecurity score was modeled continuously to preserve variability and evaluate whether increasing severity of food insecurity was associated with increasing water insecurity. Statistical significance was defined as a two-sided *p*-value < 0.05.

## 3. Results

### 3.1. Sample Characteristics

**Figure 1:**
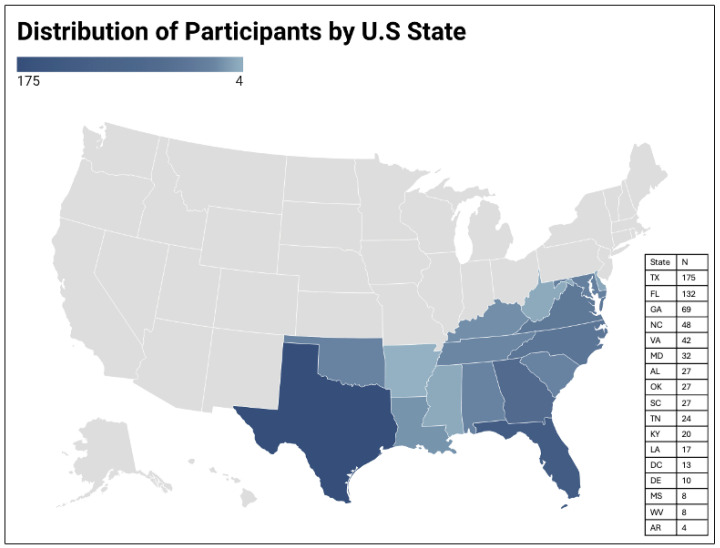
Geographic distribution of study participants across U.S. states within the Southern US region.

The analytic sample included 683 SNAP-participating adults residing in households with children in the Southern U.S. The majority of the sample were from Texas (*n* = 175) and Florida (*n* = 132). Figure 1 shows the distribution of all participating states. The sociodemographic and household characteristics of participants are detailed in Table 1. Briefly, the sample was approximately evenly distributed by gender (49.8% male; 50.1% female). The majority of respondents identified as non-Hispanic White (67.6%), followed by non-Hispanic Black (17.9%) and Hispanic/Latino (10.5%). More than half of participants (56.8%) reported having a bachelor’s degree or higher, and most were employed either full-time or part-time (82.9%). In terms of household composition, 69.3% of respondents lived in households with four or more members. Most households (81.1%) included one to two children, while 18.9% had three or more children.

### 3.2. Water Insecurity

Approximately half of respondents reported at least one water insecurity experience across the four HWISE items (52.3%), comprising 22.4% reporting occasional experiences (1–3 times) and 29.9% reporting frequent experiences (>3 times). The most frequently reported experience was worrying about insufficient water (30.9%), followed by not having enough water to drink (30.3%), disruptions to plans (29.4%), and inability to wash hands (29.1%). All items were endorsed at least once, indicating that water-related challenges were present across the sample (Table 2). Overall, these findings suggest that while water insecurity was not universal, a substantial proportion of households experienced repeated water-related disruptions.

### 3.3. Prevalence of Food and Water Insecurity

Food insecurity was highly prevalent in this sample, affecting 75.1% of households. Water insecurity was also common, with 53.9% of participants classified as water-insecure. Only 19.6% of households were both food- and water-secure. In contrast, nearly half of the sample (48.6%) experienced the co-occurrence of both food and water insecurity, representing a coexisting hardship. Among households experiencing only one form of insecurity, 26.5% were classified as food-insecure only, whereas 5.3% were water-insecure only (Table 3).

### 3.4. Association Between Food Insecurity and Water Insecurity

**Figure 2:**
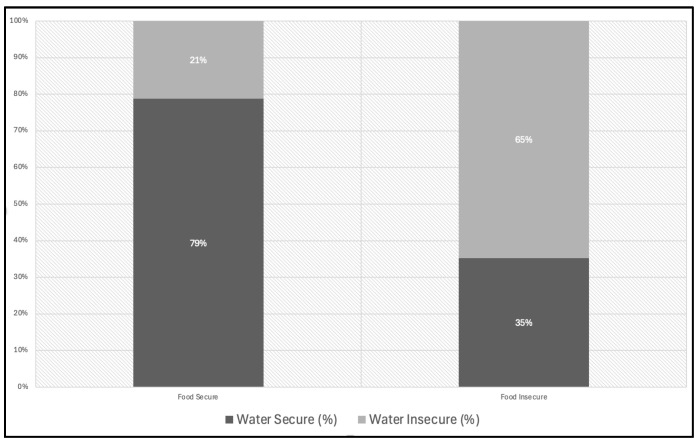
Distribution of water security status by food security category among SNAP households with children. Percentages are calculated within each food security category.

As shown in Figure 2, water insecurity was substantially more prevalent among food-insecure households compared with food-secure households. The majority of food-insecure households experienced water insecurity, whereas water insecurity was less common among food-secure households.

In logistic regression analysis, the food insecurity score was strongly associated with water insecurity. Each one-point increase in food insecurity score was associated with a 33% increase in the odds of water insecurity (OR = 1.33, 95% CI: 1.26–1.40, *p* < 0.001) (Table 4). Household size was not significantly associated with water insecurity. The number of children in the household showed a marginal association, with households with three or more children having lower odds of water insecurity compared to those with fewer children (OR = 0.64, 95% CI: 0.41–1.00, *p* = 0.050). The overall model was statistically significant (*p* < 0.001) and explained approximately 25% of the variance in water insecurity.

## 4. Discussion

This study adds to the limited U.S.-based evidence on the co-occurrence of food and water insecurity among SNAP households with children in the Southern U.S. The study’s conceptual framing was to explore the “dual burden” of resource insecurity for both food and water security, which was supported by nearly half of households (48.6%) reporting experiences of both food and water insecurity, while fewer than one in five were secure in both domains. Importantly, our findings contributes to a limited but growing body of U.S.-based evidence by applying a validated household water insecurity measure alongside a standardized food insecurity instrument, addressing a key gap identified in the literature [19]. This disparity likely reflects the targeted focus on SNAP participants, a population already identified as being at elevated risk of economic hardship and food insecurity [26]. Similarly, the high prevalence of water insecurity in this sample (53.9%) suggests that water-related challenges are present among socioeconomically vulnerable households in the U.S. [8,15,27]. These findings support the argument introduced earlier that structural inequalities, such as limited infrastructure access, financial constraints, and geographic disparities, play a central role in shaping both food and water insecurity in the U.S. [3,4,22].

Furthermore, although the HWISE scale has been widely used internationally, its application in U.S. populations remains relatively limited. Using a validated experiential measure of household water insecurity, we found that nearly half of surveyed households experienced both food insecurity and water insecurity simultaneously, highlighting a substantial dual burden that may be overlooked when these resource insecurities are examined separately. The relatively small proportion of households experiencing water insecurity alone (5.3%) suggests that water insecurity appeared less commonly in isolation in this sample and could be a broader pattern of deprivation [28,29,30]. In contrast, over one-quarter of households experienced food insecurity without water insecurity, indicating that while water insecurity is closely linked to food insecurity, it may co-occur with broader forms of material hardship in this population [15,30,31]. This pattern aligns with emerging conceptual frameworks that position water insecurity as part of a multidimensional system of resource constraints, rather than as a standalone issue [31].

The focus on households with children is particularly important in understanding the implications of these findings. Although additional socioeconomic and environmental variables may influence this relationship, these were not available and represent an area for future research. As previously stated, water plays a critical role not only in hydration but also in food preparation, sanitation, and child feeding practices [1,2,3,4,5]. Inadequate or unreliable water access may therefore have cascading effects on child health and nutrition, including increased risk of infectious disease, compromised dietary quality, and challenges related to infant feeding [21,32]. Households with children may also face higher baseline water needs, making them more vulnerable to disruptions in access. Additionally, children are more vulnerable to effects from water contamination. For example, the same concentration of lead or arsenic will have a more severe effect on a child compared to an adult. The risk of drinking water contamination is especially relevant for families that rely on private wells, where testing for contaminants is the sole responsibility of the well owners. Families with significant financial strain may have difficulty paying for tests [33]. Higher food insecurity scores were associated with substantially increased odds of water insecurity. Household composition variables were not independently associated with water insecurity in adjusted analyses; however, this finding aligns with growing evidence highlighting the critical role of water security as a determinant of health and underscores the increasing attention within public health on the broader social determinants of health as fundamental drivers of health inequities [34,35,36].

These findings are particularly salient in the context of the Southern U.S., a region characterized by higher rates of poverty, climate vulnerability, and uneven infrastructure development [23]. As noted earlier, drought, extreme weather events, and historical underinvestment in public systems may exacerbate both water and food insecurity in this region [22,23]. The high prevalence of the concurrent food and water insecurity observed in this study suggests that regional and structural factors play a significant role in shaping household-level experiences of resource insecurity. This underscores the importance of geographically targeted interventions that account for local environmental and socioeconomic conditions.

These results also have implications for measurement and research. As emphasized in the Introduction, the lack of standardized tools for assessing water insecurity in the U.S. has limited understanding of its scope and impact. By applying the HWISE scale in a U.S. context, this study demonstrates its utility for capturing household-level water challenges and highlights the importance of integrating water insecurity into broader assessments of social determinants of health [15,19]. Future research should continue to validate and refine water insecurity measures in high-income settings and explore longitudinal relationships between food and water insecurity to better understand causal pathways [23].

Several limitations should be considered when interpreting these findings. First, the cross-sectional design precludes causal inference, and the observed association between food and water insecurity should not be interpreted as a directional relationship. Second, data were collected through a web-based survey panel, which may introduce selection bias and limit generalizability to all SNAP households. Because participation required internet access and engagement with an online survey panel, households experiencing the most severe resource deprivation may have been underrepresented. Third, both food insecurity and water insecurity were assessed using self-reported measures, which may be subject to recall or reporting bias. Finally, while the HWISE scale provides a standardized measure of water insecurity, the use of a shortened version may not capture the full complexity of water-related experiences; however, it is a valid tool for measuring household water security status [20]. The educational attainment and employment characteristics of this web-panel sample differed substantially from those reported in nationally representative SNAP populations, suggesting that estimates should be interpreted as reflecting this surveyed population rather than SNAP participants broadly. In addition, the adjusted analyses were limited to available household composition variables. Other potentially important confounders, including urban/rural residence, housing tenure, water infrastructure access, financial strain, and environmental conditions, were not measured and therefore could not be accounted for, raising the possibility of residual confounding.

## 5. Conclusions

These findings provide important insights into the co-occurrence of food and water insecurity in a high-risk U.S. population. By demonstrating the extent of the dual burden among SNAP households with children, the findings underscore the need to move beyond siloed approaches to resource insecurity and toward integrated strategies that address the full spectrum of household needs. As highlighted throughout this paper, water is a fundamental but often overlooked component of food security and overall well-being. Addressing both food and water insecurity simultaneously will be critical for improving health outcomes and reducing disparities among vulnerable populations [16]. From a policy and public health perspective, the results highlight the need for more integrated approaches to addressing resource insecurity. Current federal nutrition assistance programs, such as SNAP, are designed to improve food access but do not explicitly address water access or affordability. The substantial co-occurrence of food and water insecurity observed in this analysis highlights the importance of considering multiple dimensions of resource insecurity when assessing household vulnerability. Future research should examine how water access and affordability intersect with food insecurity and evaluate whether integrated approaches to addressing these challenges may improve household well-being [37,38]. Additionally, incorporating measures of water insecurity into routine surveillance and program evaluation could improve identification of at-risk populations and inform more effective resource allocation.

## Figures and Tables

**Table 1:** Sociodemographic and household characteristics of SNAP participants residing in households with children (N = 683).

Characteristic	Category	*n* (%)
Gender	Male	340 (49.8)
	Female	342 (50.1)
Race/Ethnicity	Non-Hispanic White	462 (67.6)
	Non-Hispanic Black	122 (17.9)
	Hispanic/Latino	72 (10.5)
	Other	27 (4.0)
Education	High school or less	166 (24.3)
	Some college/associate	129 (18.9)
	Bachelor’s or higher	388 (56.8)
Employment	Employed (full/part-time)	566 (82.9)
	Unemployed	77 (11.3)
	Other	40 (5.8)
Household Size	1 person	10 (1.5)
	2–3 persons	200 (29.3)
	≥4 persons	473 (69.3)
Children in Household	1–2 children	554 (81.1)
	≥3 children	129 (18.9)

Note: Percentages may not total 100 due to rounding.

**Table 2:** Frequency of HWISE-4 water insecurity experiences in the previous 4 weeks.

Experience	0 Times (%)	1–3 Times (%)	>3 Times (%)
Worry about not having enough water	45.3	23.8	30.9
Had to change plans due to water problems	48.7	21.9	29.4
Not enough water to drink as preferred	47.1	22.6	30.3
Unable to wash hands due to water problems	49.5	21.4	29.1
Average across experiences	47.7	22.4	29.9

Note: Exact denominator varies slightly per item due to exclusion of “Not applicable/Don’t know” among N = 683.

**Table 3:** Prevalence of food insecurity, water insecurity, and dual burden.

Outcome	*n* (%)
Food Insecurity Status	
Food-secure	170 (24.9)
Food-insecure	513 (75.1)
Water Insecurity Status	
Water-secure	315 (46.1)
Water-insecure	368 (53.9)
Co-Occurrence (Dual Burden Categories)	
Food- and water-secure	134 (19.6)
Food-insecure only	181 (26.5)
Water-insecure only	36 (5.3)
Both food- and water-insecure	332 (48.6)

Note: Percentages are based on the total sample. Food insecurity was classified using the USDA 10-item module; water insecurity was assessed using the HWISE scale. Categories are mutually exclusive.

**Table 4:** Multivariable logistic regression: association between food and water insecurity.

Variable	AOR	95% CI	*p*-Value
Food insecurity score	1.33	1.26–1.40	<0.001
Household size (2–3 vs. 1)	0.58	0.14–2.50	0.468
Household size (4+ vs. 1)	0.72	0.17–3.00	0.647
Children (3+ vs. 1–2)	0.64	0.41–1.00	0.050

Note: OR = odds ratio; CI = confidence interval. Food insecurity score is continuous. Reference groups: household size = 1 person; children = 1–2. Outcome: moderate/high vs. low/no water insecurity.

## Data Availability

The participant-level data are not publicly available because they contain potentially identifying information from human participants. Requests for access may be directed to the corresponding author and will be considered subject to institutional and ethical approvals.
